# A missense mutation in *PDHB* gene: identification of the patient with pyruvate dehydrogenase deficiency and demonstration of pathogenicity in vitro

**DOI:** 10.1186/s13052-025-01917-9

**Published:** 2025-03-06

**Authors:** Ying Li, Lin Cheng, Xin Li, Jiyu Liu, Lu Yi, Tao Bo

**Affiliations:** 1 Division of Neonatology, Department of Pediatrics, The Third Xiangya Hospital of Central South University, Changsha, Hunan China; 2 Children Medical Center, The First Affiliated Hospital of University of South China, Hengyang, Hunan China

**Keywords:** PDH, Pyruvate dehydrogenase deficiency, E1 β subunit, *PDHB* gene, Mutations, Lactic acidosis

## Abstract

**Background:**

Pyruvate dehydrogenase (PDH) deficiency is an uncommon condition responsible for primary refractory lactic acidosis, and PDH E1β (*PDHB*) subunit gene mutation rarely causes of PDH deficiency. We described a missense mutation of *PDHB* gene in a neonate with PDH deficiency, and verified the mutation damages PDH activity in vitro.

**Methods:**

Whole exome sequencing (WES) was used to discover the missense mutation. We constructed the recombinant eukaryotic recombinant expression vector, the phage-PDHB-wt/mut, containing human full-length wild-type (NM_000925.4) or mutant (c.575G > T) *PDHB* gene, and transfected vector into 293T cells. Western blot was performed to assess PDH protein stability, PDH activity was measured.

**Results:**

A 37-week-gestation male infant was noted to have refractory lactic acidosis, growth retardation, and neurodevelopmental anomalies with abnormal brain magnetic resonance (MR) findings, starting with convulsive seizures at 3 months of age. WES analysis revealed the homozygous missense mutations in the *PDHB* gene, which was c.575G > T (p.Arg192Leu) in exon 6. This missense mutation of *PDHB* was predicted to be harmful by bioinformatics software including Sorting Intolerant From Tolerant (SIFT), Polyphen2, LRT, and Mutation Taster. Western blot showed that normal PDH protein expression was significantly decreased in the phage -PDHB-mut transfected cells than that in the phage -PDHB-wt transfected cells (*P* < 0.001). PDH activities analysis revealed that PDH activity was significantly decreased in the phage -PDHB-mut transfected cells than that in the phage -PDHB-wt transfected cells (*P* < 0.001).

**Conclusions:**

c.575G > T (p.Arg192Leu) in *PDHB* gene is a pathogenic missense mutation, which causes PDH deficiency in autosomal recessive inheritance mode.

## Background

Pyruvate dehydrogenase (PDH) complex is the key enzyme for mitochondrial oxidative metabolism of carbohydrate, catalyzing the irreversible decarboxylation of pyruvate and formation of acetyl-CoA and NADH [1–3]. PDH deficiency is a common cause of primary lactic acidosis. Clinical consequences primarily affect the nervous system, including developmental delay, seizures, central hypotonia, microcephaly, congenital brain malformations, and degenerative changes such as Leigh syndrome [2, 4, 5]. Blood and/or cerebrospinal fluid (CSF) lactate are usually increased, with a normal lactate to pyruvate ratio and increased alanine [1].

PDH complex comprises three catalytic components, including pyruvate dehydrogenase (E1), dihydrolipoamide acetyltransferase (E2) and dihydrolipoamide dehydrogenase (E3). The E1 enzyme is a symmetric heterotetramers composed of two α and two β subunits (αα'ββ') that binds two molecules of thiamine pyrophosphate (TPP) as a cofactor [6]. Most commonly, PDH deficiency is due to mutations of *PDHA1* gene for the E1α subunit located on chromosome X. To date, over 100 mutations of *PDHA1* have been reported [7, 8]. In contrast, rarely cases with mutations of the *PDHB* gene for the E1β subunit, which maps to chromosome 3, had been previously reported [8, 9]. Here we report a Chinese newborn infant with PDH deficiency, to the best of our knowledge, caused by a pathogenic missense mutation in *PDHB* gene, and verify that the mutation damages PDH activity in vitro.

## Methods

### Case

A Chinese newborn infant with refractory lactic acidosis carried *PDHB* missense variants. The clinical manifestations and gene variations were analyzed.

### Variation analysis

Written informed consent was obtained from the parents of the patient. 5 mL of peripheral venous blood of the infant, his parents and his brothers were collected. The transfected cells were extracted, and genomic DNA was extracted for next-generation whole exome sequencing (WES) of family trio. This study was approved by the institutional review board of the Third Xiangya Hospital of Central South University.

### Pathogenicity identification of the missense mutation

#### Vector construction and transfection

The recombinant plasmid vector pEGFP-N1 containing human full-length wild-type (NM_000925.4) or mutant (c.575G > T) *PDHB* were constructed by Cipher Decoding (Beijing, China) Gene Technology Co., Ltd. To obtain the phage-PDHB-wt/mut, the PEGFP-N1-PDHB- wt/mut cDNA sequence was cloned into the phage with restriction enzymes SalI and BamH I. A pair of primers were designed: BamHI tailed forward (5′-CGCTGGATCCATGGCGGCGGTGTCTGGCTTGGT-3′) and salI tailed reverse (5′-CGAGGTCGACGGAATATTTAATGTTTTCTTTA-3′) primers. The primers were synthesized and desalted from Cipher Decoding Gene Technology Co., Ltd. The recombinant vector phage-PDHB-wt/mut was further sequenced to confirm its sequence by Cipher Decoding Gene Technology Co., Ltd.

According to the manufacturer’s instructions, transfection with the constructed wild-type or mutant eukaryotic recombinant expression vector phage -PDHB-wt/mut into 293 T cells (Thermo Fisher Scientific, R70007, America) was performed using Lipofectamine 2000 (Thermo Fisher Scientific, 11668019, America). The E1β subunit expression was evaluated by quantitative real-time PCR (qPCR) or western blot analysis at 48 h after transfection.

#### qPCR assay

Total RNA was extracted using a TRIzol Plus RNA purified Kit (Invitrogen, 12,183,555, America) according to the manufacturer’s instructions. First-strand cDNA was then synthesized from 1 µg of RNA using the EvoM-MLV RT for PCR Kit (Accurate Biology, AG11603, China). qPCR was performed using TB Green Premix Ex Taq II (Takara, RR820Q, Japan) on the ABI 7500 Real-Time PCR System (Thermo Fisher Scientific, America). Analysis was performed using ABI 7500 software. The data were normalized to RNA GAPDH and the fold change was calculated via the 2-DDCt method. The relative concentrations of mRNA were expressed in arbitrary units based on the untreated group, which was assigned a value of 1. The primers, which were synthesized and desalted, were from Cipher Decoding Gene Technology Co., Ltd.

#### Western blot analysis

To assess mutant protein stability, the transfected cell were extracted with cell lysis buffer (Beyotime, P0013, China). Lysates were centrifuged and supernatants were collected. Protein samples were resolved by SDS-PAGE and transferred to Polyvinylidene fluoride membranes (Millipore, IPVH00010, America) in a semidry transfer system (Bio-Rad, America). Membranes were blocked with 5% BSA for 1 h at room temperature and subsequently immunoblotted with the antibodies [rabbit monoclone antibody against PDH (1:1000, CST, 2784, America), mouse monoclone antibody against β-actin (1:5000, Proteintech, 66,009–1-Ig, China)] overnight followed by horseradish peroxidase- conjugated secondary antibody [goat polyclone antibody against mouse IgG (1:1000, Proteintech, SA00001-1, China)and goat polyclone antibody against rabbit IgG (1:1000, Proteintech, SA00001-2, China)]. Blots were analyzed by digital gel imaging analysis system (Invitrogen, America).

#### PDH activity measurement

Transfected 293T cells were collected at 48 h after transfection. According to the manufacturer’s instructions, PDH activity of these transfected cells was measured with a commercial enzyme assay kit (Solarbio Science & Technology, BC0385, America).

### Statistical analysis

SPSS 20.0 was used to collect and analyze data. The statistical analyses used in this study are indicated in the respective figure legends. Data with two groups were analyzed by Student unpaired *t*-test to determine statistically significant effects.

## Results

### Case presentation

A 37-week-gestation male infant was born to a 29-year-old gravida 3, para 3, woman via Cesarean section for oligohydramnios. His birthweight was 2100 g (< 3rd percentile). The infant was vigorous at birth. Due to refractory lactic acidosis, the patient was transferred to neonatal intensive care unit (NICU) at 8 day after birth. His parents are not related by blood. The mother had no antenatal risk factors. And part of the prenatal investigations and findings was missing because the mother gave birth outside hospital. There was no significant familial history of congenital abnormalities. His parents and two old brothers were healthy.

When he was admitted to NICU, his birthweight was 2.05 g (< 3rd percentile), and head circumference 29 cm (< 3rd percentile). He presented slight tachypnea, but no cyanosis. The remainder of the documented physical examination findings were unremarkable. Arterial blood gas measurement showed compensated metabolic acidosis and increased lactic acid (Table 1). Blood routine, urine routine, stool routine, liver function and renal function testing were normal. No hypoglycemia, hyperammonemia or electrolyte abnormality is noted. The abnormal type of brainstem auditory evoked potential was central hearing damage of the brain stem.

**Table 1:** Arterial blood gas during admission

	pH	PaCO_2_ (mmHg)	PaO_2_ (mmHg)	Bicarbonate (mmol/L)	BE (mmol/L)	Lactate acid (mmol/L)
Day 0	7.33	35	51	-	−11.8	-
Day 2	7.37	35	63	-	−14.3	-
Day 3	7.32	18	97	12.8	−15.2	12.6
Day 7	7.39	28	61	18.7	−6.7	7.9
Day 9	7.41	16	149	13.8	−13.5	8.4
Day 14	7.38	30	78	19.1	−6.5	7.6
Day 21	7.41	23	106	14.6	−8.5	6.8

*PaCO*_*2*_ Partial pressure of arterial carbon dioxide, *PaO*_*2*_ Partial pressure of arterial oxygen, *BE* Base Excess

Blood organic acid analysis suggested pyruvate level of 792.99μmol/L (normal < 400μmol/L). Urine organic acid analysis suggested ketonuria, with pyruvate level of 72μmol/L (normal < 30μmol/L), 2-hydroxybutyric acid level of 4.4μmol/L (normal < 2μmol/L) and 3-hydroxybutyric level of 15.1μmol/L (normal < 9μmol/L).

**Fig. 1:**
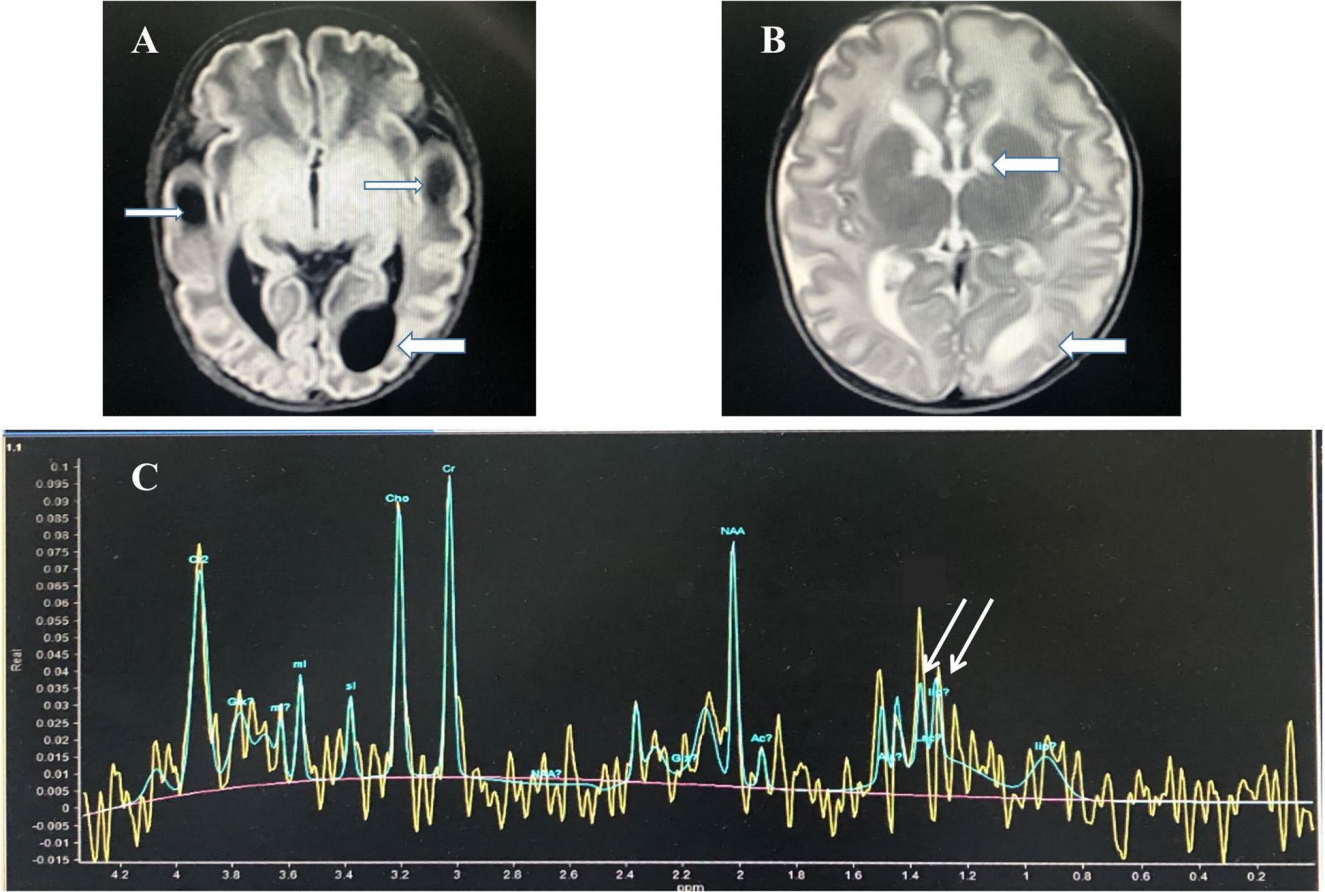
Brain magnetic resonance imaging on the 18d after birth. **A** showed symmetric decreased T1 signal in temporal lobal white matter (thin arrow) and subependymal cyst (thick arrow) on T1 weighted image; **B** showed multiple subependymal cysts (thick arrow) on T2 weighted image; **C** showed lactate doublet was present at 1.3 ppm (arrow) on single-voxel proton MR spectroscopy obtained with time of echo (TE) of 35 ms

Ultrasonic examination revealed that heart, liver, spleen, and kidney were normal. Brain magnetic resonance (MR) was performed on the 18d after birth. MR image showed diffused decreased T1 signal and increased T2 signal in cerebral white matter, increased T1 signal and decreased T2 signal in thalamus and basal ganglia with multiple subependymal cysts and dysplasia of corpus callosum. On single-voxel proton MR spectroscopy obtained with TE of 35 ms, lactate doublet was present at 1.3 ppm (Fig. 1).

After admission, the infant was supplied sufficient energy and treated with sodium bicarbonate supplementation to correct metabolic acidosis, but he continued to present inconsistent weight gain, and lactic acidosis (Table 1). The neonate was discharged on day 21 after birth. Seizures began to appear 3 months after birth. After using ketogenic diet, seizures were relieved. Presently he is 16 months old, undergoing follow-up evaluations, his body weight was 6.2 g (< −3SD). His current developmental age is only 3 months with global developmental delay and hearing loss being noted.

### Molecular genetic analysis

**Fig. 2:**
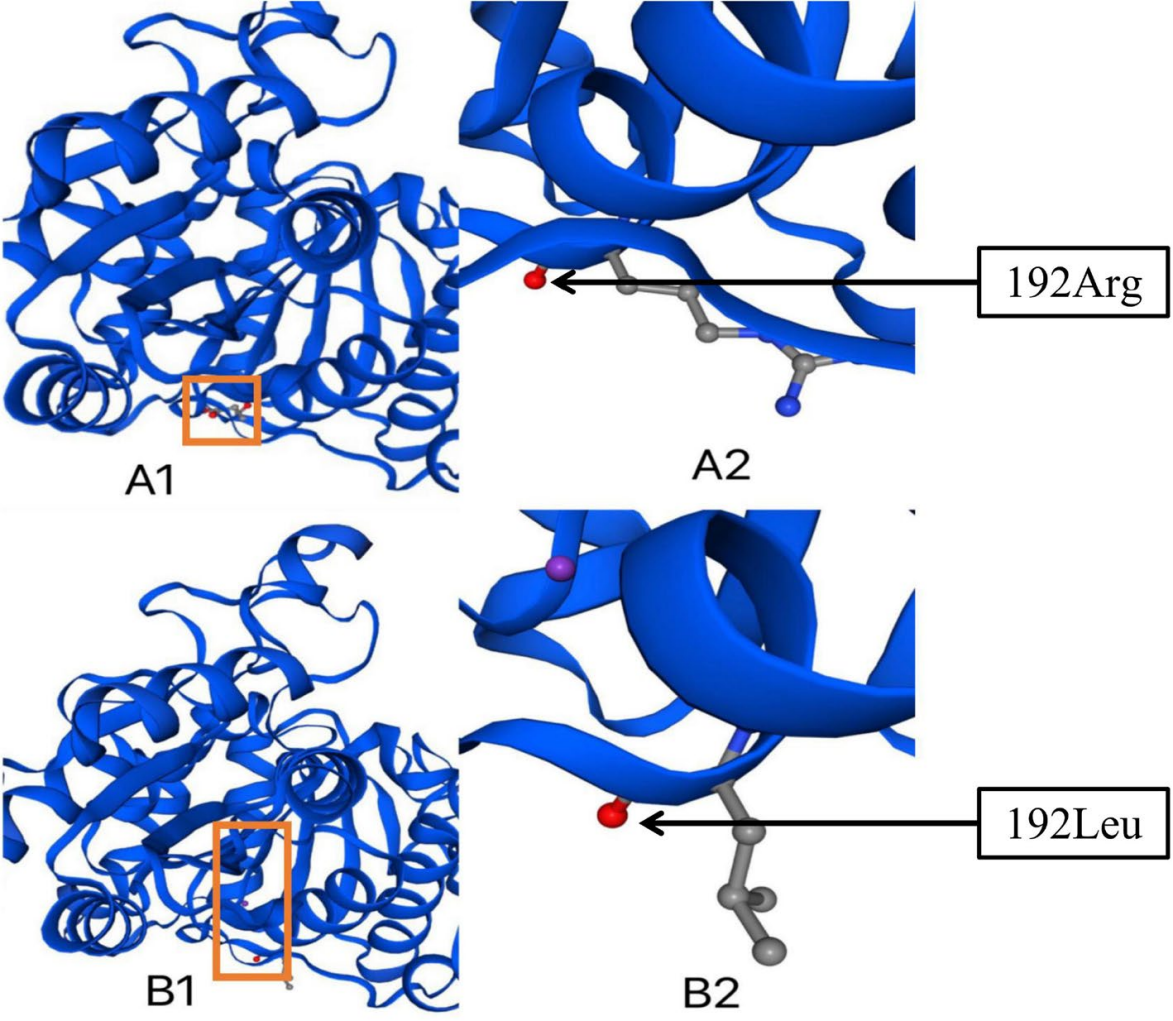
Three-dimension homology model changes induced by the c.575G > T mutation in PDH E1 β subunit. A1 and A2: wild type; B1 and B2: c.575G > T (p.Arg192Leu) mutation

**Fig. 3:**
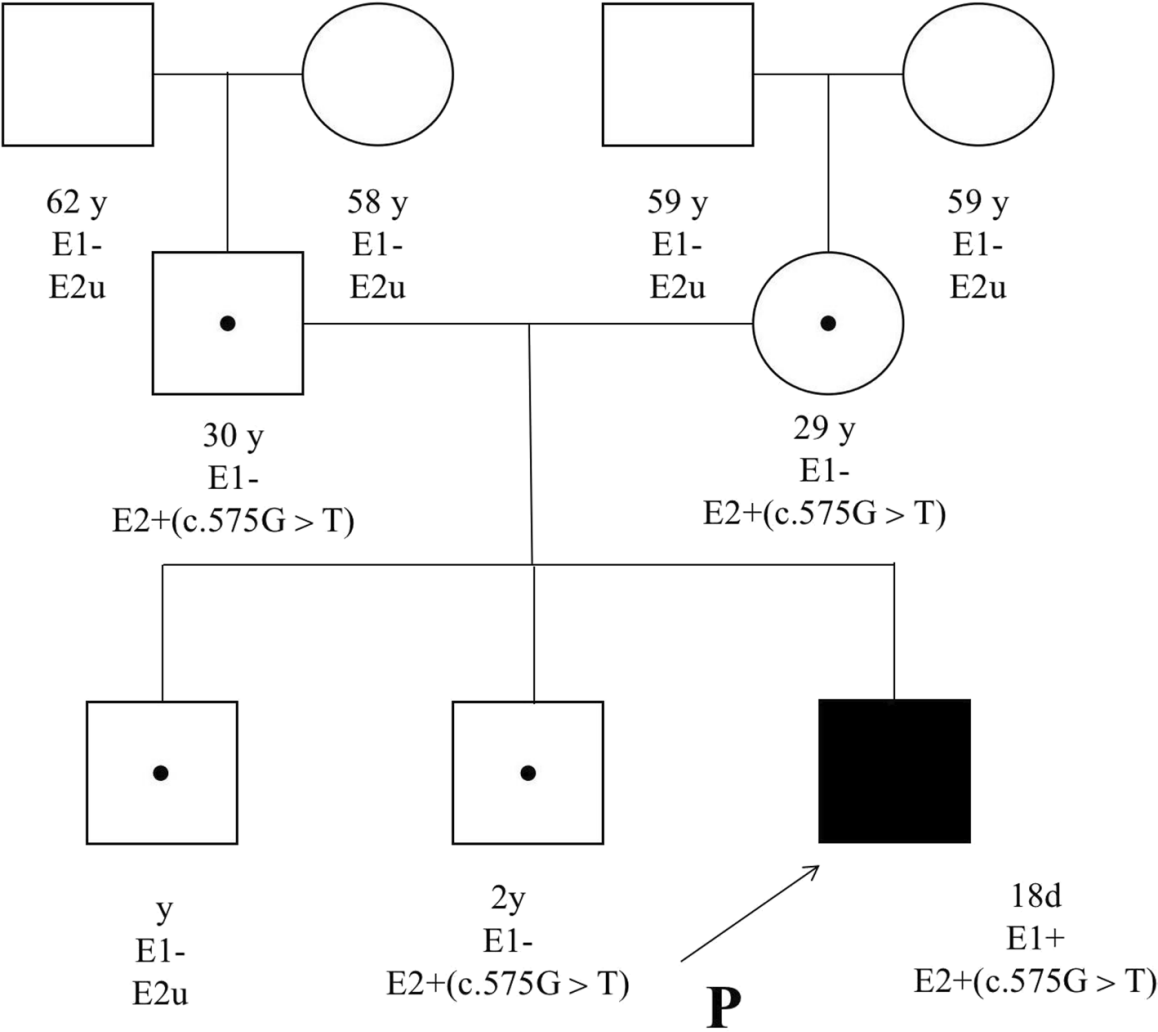
The patient’s family pedigree. The c.575G > T mutation in PDH E1 β subunit was found in *PDHB* gene of this proband. The patient’s parents, each of whom carries a point mutation in the c.575G > T mutation in PDH E1 β subunit. The patient’s brothers are the same carrier. E1, physical examination; E2, whole-exome next-generation sequencing; P, proband

**Fig. 4:**
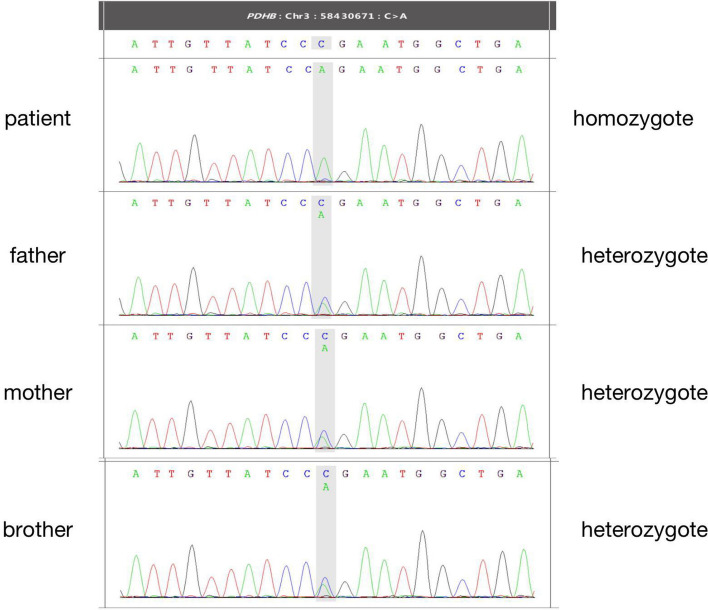
The sequencing of the Trio

Whole exome sequencing (WES) analysis revealed the homozygous missense mutations in the *PDHB* gene, which was c.575G > T (p.Arg192Leu) in exon 6 (Fig. 2), and no other gene mutations related to hyperlactatemia were found. His parents and brother were heterozygotes with the same mutation in *PDHB* gene (Figs. 3 and 4).

In the Genome Aggregation Database (gnomAD), Exome Aggregation Consortium (ExAC_ALL), and Exome Aggregation Consortium East Asian (ExAC_EAS), no information of the frequency of the mutations in normal population were found. Furthermore, mutation pathogenicity prediction software, including Sorting Intolerant From Tolerant (SIFT), Polyphen2, LRT, MutationTaster, and PredictSNP (https://loschmidt.chemi.muni.cz/predictsnp/), predicted that the mutation of c.575G > T is harmful and destabilizing. The value of REVEL is 0.835.

The SWISS-MODEL workspace (http://swissmodel.expasy.org) was used to characterize the effect of the mutation on the E1βsubunit protein. Homology modeling of the amino acid was employed as the template of the Protein Data Bank (PDB) structure. We found that the structures of the protein resulting from the mutation of c.575G > T were predicted and compared with that of the wild type, whereupon significant 3D homology model differences were observed (Fig. 2). The predicted structure of the splice site mutation c.575G > T (p.Arg192Leu) in exon 6 revealed a significant abnormal structure of the protein that might cause protein dysfunction.

**Fig. 5:**
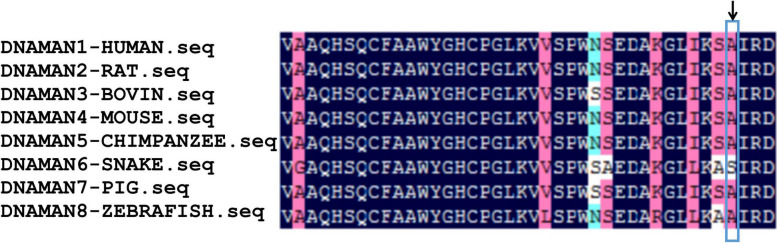
Evolutionary conservation of the mutated residue in PDH E1β subunit. Arrow points to position 192 in PDH E1β subunit. HUMAN, Homo sapiens (Human); RAT, Rattus norvegicus (Rat); BOVIN, Bos taurus (Bovine); MOUSE, Mus musculus (Mouse); CHIMPANZEE, Pan troglodytes (chimpanzee); SNAKE, Notechis scutatus (mainland tiger snake); PIG, Sus scrofa (pig); ZEBRAFISH, Danio rerio (zebrafish)

Sequence alignments among eight species suggested that arginine at position 192 is highly conserved in PDH E1 β subunit (Fig. 5).

### Generation and expression of the mutant cDNA

**Fig. 6:**
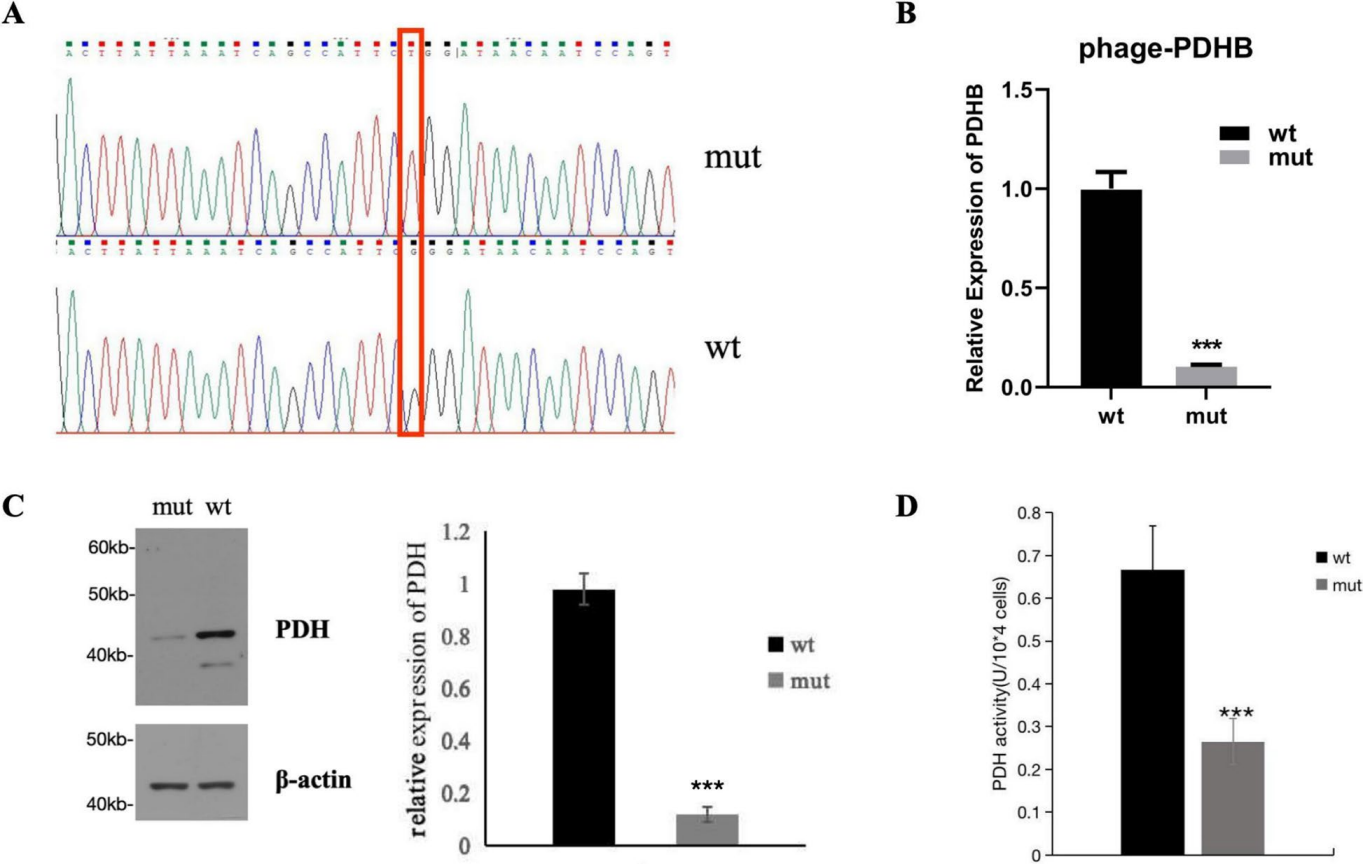
**A **CDNA sequencing analysis revealed that the c.575G > T nucleotide mutation was introduced into phage -PDHB-mut. **B** *PDHB* mRNA expression in wild-type transfected cells and in mutant transfected cells (qPCR). **C** PDH protein expression in wild-type transfected cells and in mutant transfected cells. **D** PDH activity were measured in wild-type transfected cells and in mutant transfected cells. The data is representative of three independent experiments in qPCR, western blot, and PDH activity measurement. Data were presented as mean ± SD. Statistical significance was analyzed using two-way ANOVA with Dunnett’s multiple comparisons test. ***: compared to wild-type transfected cells, *P* < 0.001

CDNA sequencing analysis revealed that the c.575G > T nucleotide mutation was introduced into phage -PDHB-mut, and confirmed the absence of additional mutations in phage -PDHB-mut (Fig. 6A). qPCR showed normal *PDHB* mRNA expression was significantly decreased in the phage -PDHB-mut transfected cells than that in the phage -PDHB-wt transfected cells (*P* < 0.001) (Fig. 6B).

Western blot showed that normal PDH protein expression was significantly decreased in the phage -PDHB-mut transfected cells than that in the phage -PDHB-wt transfected cells (*P* < 0.001) (Fig. 6C). PDH activities analysis revealed that PDH activity was significantly decreased in the phage -PDHB-mut transfected cells than that in the phage -PDHB-wt transfected cells (*P* < 0.001) (Fig. 6D).

## Discussion

PDH deficiency, with its diverse and non-specific clinical manifestations, poses a challenge for clinical diagnosis. Clinically PDH deficiency shows a wide range of variations from fatal lactate acidosis and brain abnormalities, to epilepsy and neuromuscular dysfunction with neurodevelopmental delay [10–12]. In our case, the patient was only noted to have intrauterine growth retardation, oligohydramnios and refractory lactate acidosis during the perinatal period. Subsequently, the patient experienced growth retardation and neurological abnormalities, starting with convulsive seizures at 3 months of age, abnormal brain MR findings and hearing loss. None of these manifestations can unequivocally diagnose PDH deficiency.

The diagnosis of PDH deficiency is often difficult only based on plasma amino acid quantification assay and urine organic acid quantification assay. Therefore, special assay method has been established to measure PDH activity in muscle and skin fibroblasts for diagnosis [13]. However, it has been found that due to the tissue specificity of the decreased PDH activity in the patient, some patients with PDH deficiency only experience severe decreases in enzyme activity in organs such as the heart, brain, and liver [14]. Therefore, genetic testing is a reliable and effective method for the diagnosis of PHD deficiency.

The human PDH E1 exists in the form of *α2β2*, which has been confirmed by crystallographic studies. *PDHA1* gene is located at Xp22.12, which is 17 kb and contains 11 exons. Since the serine site of PDH E1 *α* subunit plays an important role in regulating the entire PDH complex activity, the mutations in *PDHA1* gene are the most common in PDH deficiency [7]. To date, there are more than 150 pathogenic mutations in the *PDHA1* gene, which are distributed throughout *PDHA1* gene, including missense, nonsense, splice, insertions and deletions mutations. Most missense mutations are located in exons 1–9, which can easily lead to loss of PDH complex activity, while most frameshift mutations are located in exons 10 and 11, which altered expression of PDH E1α mRNA and protein [1] (http://www.hgmd.cf.ac.uk/ac/index.php).

*PDHB* gene is located at 3p14.3, with a total length of 1.5 kb and 10 exons. In PDH deficiency cases, mutations in *PDHB* gene are significantly less common than those in *PDHA1* gene, accounting for only a small proportion of PDH deficiency cases [10, 15–17]. In 2004, Brown et al. first reported the mutation of *PDHB* gene in two patients with PDH deficiency [18]. In a retrospective study of 588 reported PDH deficiency cases from 1970 to 2014, only 20 cases were caused by mutations in *PDHB* gene [6].

## Conclusions

In our case, WES analysis revealed the homozygous mutations in exon 6 of the *PDHB* gene, c.575G > T (p.Arg192Leu). The same mutation was first reported in April 2023, but the clinical significance of this mutation remains unclear [19]. We used bioinformatics to predict that the mutation of c.575G > T was harmful and destabilizing, leading to the change of the spatial structure of the PDHc protein. The evolutionary conservation of the arginine 192 residue in PDH E1 β subunit and the invariance of this residue in the eight species (HUMAN, RAT, BOVIN, MOUSE, CHIMPANZEE, SNAKE, PIG, ZEBRAFISH) also suggest an important contribution to PDH function. Functional testing in vitro demonstrated that this is a loss-of-function mutation, with destruction of PDHc structural stability. Therefore, this mutation, c.575G > T (p.Arg192Leu), is pathogenic. His parents and brother, who did not show abnormalities as heterozygotes, indicate an autosomal recessive inheritance pattern. It has been reported the role of consanguinity and the detrimental effects of gene variants in case of autosomal recessive mode of inheritance in some research [20].

We stress the relevance of genetic testing within the clinical practice. It is useful not only in avoiding invasive investigations (moreover potentially fruitless tests for possible mosaicism and different distribution within cells and organs of mutations), but also for the possibility to provide adequate counselling to families, suggest genotype–phenotype correlations and prospect individualized management and follow-up [21–25]. In summary, c.575G > T (p.Arg192Leu) in *PDHB* gene is a pathogenic missense mutation, which causes PDH deficiency in autosomal recessive inheritance mode.

## Data Availability

The raw data supporting the conclusions of this article will be made available by the authors, without undue reservation, to any qualified researcher.

## Abbreviations

CSF: Cerebrospinal fluid
MR: Magnetic resonance
NICU: Neonatal intensive care unit
PDH: Pyruvate dehydrogenase
PDHB: PDH E1β
PDB: Protein Data Bank
SIFT: Sorting Intolerant From Tolerant
TPP: Thiamine pyrophosphate
WES: Whole exome sequencing
